# Complicated Mucocutaneous Leishmaniasis With Superimposed Cellulitis in an Immunocompromised Patient: A Case Report

**DOI:** 10.7759/cureus.71124

**Published:** 2024-10-09

**Authors:** Hussein Taleb, Islam Mukhtar, Abdulrahman H Alashkar, Mohamed I Hassan, Ahmed Alhumidi

**Affiliations:** 1 Department of Internal Medicine, Dr. Sulaiman Al-Habib Medical Group, Buraidah, SAU; 2 Department of Surgery, Dr. Sulaiman Al-Habib Medical Group, Buraidah, SAU; 3 Department of Pathology, King Saud University, Riyadh, SAU

**Keywords:** cellulitis, immunocompromised, leishmania amastigotes, mucocutaneous leishmaniasis, synovial leishmaniasis

## Abstract

Leishmaniasis is a common protozoal infection that could be cutaneous (CL), mucocutaneous (MCL), or visceral. CL, which is the most common form, is typically localized. Therefore, it becomes more difficult to diagnose it when presenting with diffuse lesions. In this case, a 54-year-old man presented with skin lesions involving his trunk, extremities, and face, including the nasal mucosa. His past medical history was remarkable for MCL with synovial leishmaniasis, systemic lupus erythematosus (SLE), and non-Hodgkin’s lymphoma (NHL). Skin biopsies showed intracytoplasmic leishmania amastigotes; polymerase chain reaction (PCR) was positive for leishmania DNA; and a culture from purulent skin lesions grew Pseudomonas aeruginosa. So, MCL with superimposed cellulitis was diagnosed, and the patient was treated with intravenous liposomal amphotericin B and ceftazidime. Leishmaniasis is an infection that has accurate diagnostic tests and various treatment options. However, the difficulty is in being able to suspect it clinically, as it can mimic a wide range of diseases with cutaneous involvement. Therefore, visual awareness of the spectrum of disease presentations is arguably the most challenging and important skill to acquire in the diagnosis and management of CL. This case represents a rare form of MCL.

## Introduction

Leishmaniasis is a common protozoal infection affecting up to one million new individuals every year. Twenty species have been implicated in human disease, causing infections in 98 countries [1]. It is transmitted through sandfly bites and can present as cutaneous (CL), mucocutaneous (MCL), or visceral disease [2]. CL, which is the most common form, is typically localized, yet the resulting lesions can have different appearances, such as eczematoid, hyperkeratotic, sporotrichoid, and plaque-like, among others [3]. Thus, these lesions have a broad differential and can mimic a wide range of dermatologic conditions or cutaneous manifestations of systemic diseases. This is especially important when leishmaniasis patients present with multiple lesions, as a high index of suspension is required for the diagnosis [4]. In this case, we discuss a rare presentation of this common disease. 

## Case presentation

A 54-year-old man presented with a three-month history of painless skin lesions involving his trunk, extremities, and face. The lesions on the extensor surface of the distal right forearm and dorsum of the right hand were painful. He did not complain of pruritus and did not report any history of fever.

His past medical history was remarkable for MCL with synovial leishmaniasis involving his ankles, right wrist, and metacarpophalangeal and interphalangeal joints, which was diagnosed and treated two years earlier at another facility. He was on miltefosine secondary prophylaxis, which he stopped four months prior to his current presentation due to gastrointestinal side effects. He is known to have chronic kidney disease and systemic lupus erythematosus (SLE) and was taking prednisolone 2.5 mg orally once daily. Moreover, he had a history of non-Hodgkin’s lymphoma (NHL) and splenectomy due to multiple splenic abscesses over 20 years ago.

**Figure 1:**
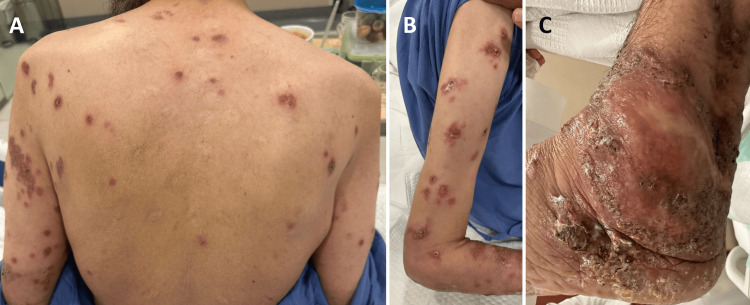
Erythematous nodular skin lesions that are ulcerated and crusted over the trunk (A), extremities (B), and left ankle (C).

When examined, the patient was afebrile and hemodynamically stable. He was neither pale nor jaundiced. Extensive erythematous nodular skin lesions that were ulcerated and crusted were noted over the trunk and extremities (Figure 1).

**Figure 2:**
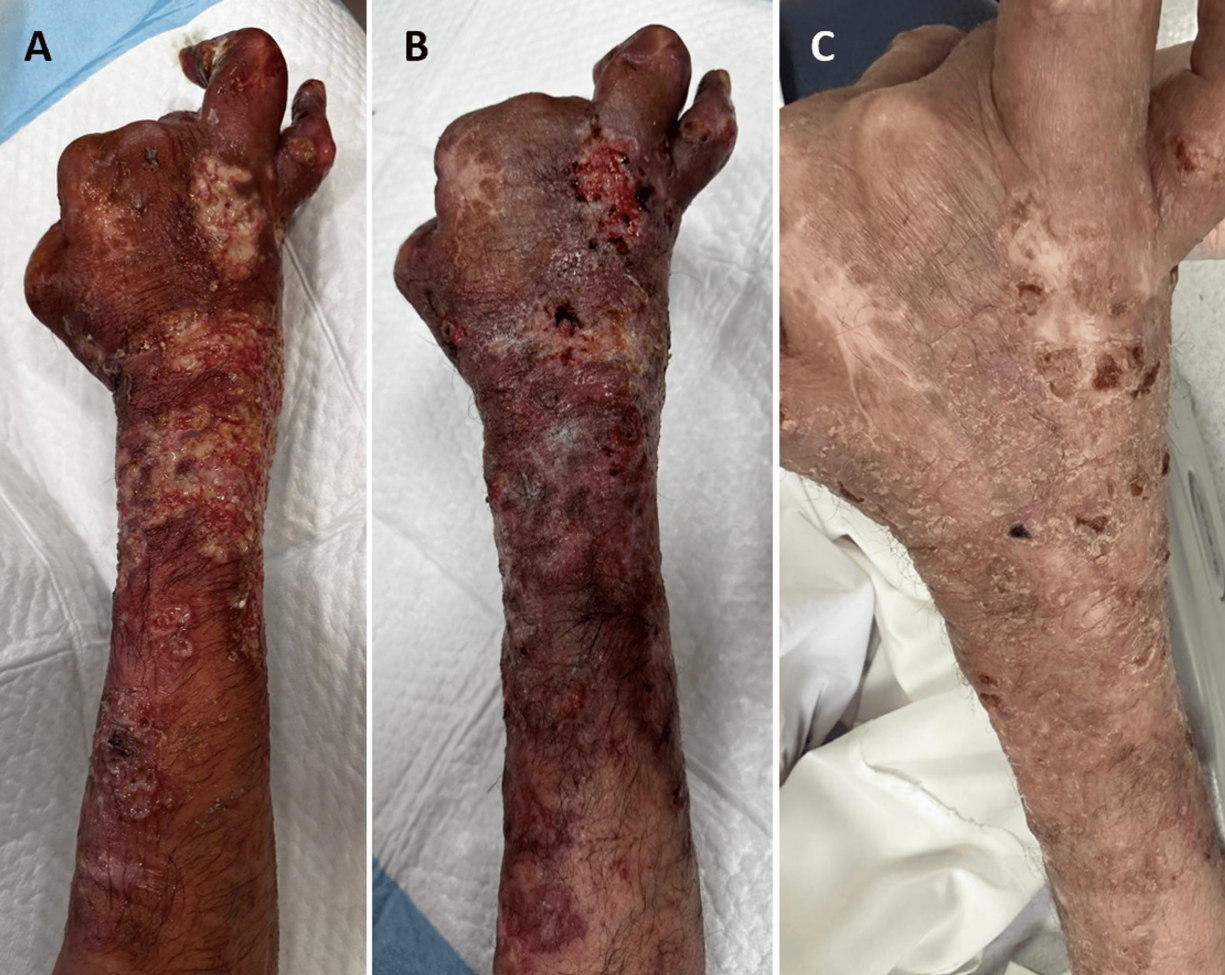
The extensor surface of the right forearm and dorsum of the right hand. A: at presentation, the lesions in this area were purulent; B, C: show the same area two weeks and two months later, respectively. In Figure 2B, the lesions started to re-epithelize. In Figure 2C, they became dry and scaly.

Also, lesions were noted on the right cheek and nose, with superficial involvement of the nasal mucosa. The lesions involving the distal right forearm were purulent (Figure 2). Right hand and fingers and left ankle deformities were noted, which the patient reported as chronic sequelae of his prior synovial leishmaniasis. The ophthalmologic exam revealed no uveitis, and the rest of the systemic examination was unremarkable. Specifically, he had no lymphadenopathy or abdominal organomegaly.

Blood investigations revealed normocytic anemia and elevated C-reactive protein and serum creatinine levels. His white blood cell count and neutrophil differential were within normal. The HIV combination test was negative (laboratory values are listed in Table 1).

**Table 1:** Laboratory values at the time of the patient's presentation and a month later.

Test	Test results upon admission	Test results a month later	Reference range
White blood cell count	6,500 cells/μL	6,660 cells/μL	4,000-11,000 cells/μL
Neutrophils %	53%	46%	37 – 80%
Red blood cells	3.36 million cells/μL	4.1 million cells/μL	4.04 – 6.13 million cells/μL
Hemoglobin	9.5 g/dL	11.2 g/dL	13 – 17.4 g/dL
Mean corpuscular volume	87 fl	80 fl	78 – 96 fl
C-reactive protein	110 mg/L	17 mg/L	˂ 5 mg/L
Serum creatinine	260 µM/L	232 µM/L	64 – 104 µM/L

**Figure 3:**
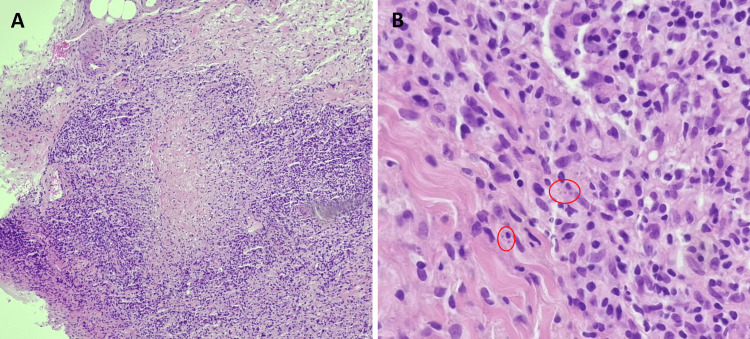
Microscopic images of the skin biopsy. A: skin biopsy of a lesion showing a necrotizing granuloma (original magnification x40, hematoxylin and eosin staining); B: A x400 magnification showing the intracytoplasmic leishmania amastigotes (red circles).

Two skin biopsies were taken from the margin of the right forearm lesions and showed necrotizing granuloma and intracytoplasmic leishmania amastigotes (Figure 3). PCR was positive for leishmania DNA. Additionally, a swab culture from the purulent skin lesions grew Pseudomonas aeruginosa. Thus, the patient was diagnosed with MCL and superimposed cellulitis involving the lesions at the distal right upper extremity.

He was started on intravenous liposomal amphotericin B (received a total cumulative dose of 28 mg/kg over 10 days) and ceftazidime 2 grams intravenously once daily for a total of 14 days to treat his MCL and bacterial cellulitis, respectively. Additionally, the purulent lesions were treated with topical fusidic acid ointment. The patient was improving on therapy, and the skin lesions gradually healed; they became dry, then scaly (Figures 2B, 2C). The patient was advised to continue on suppressive therapy (liposomal amphotericin B 3 mg/kg every three weeks). However, he opted to follow at another facility. 

## Discussion

Leishmaniasis is a protozoal infection that is transmitted via infected female sandflies. It can present as CL, MCL, or visceral disease. CL is the most common form, and up to one million new cases are estimated to occur annually [2]. Synovial involvement is more documented with canine leishmaniasis. However, it has also been reported in humans [5]. In this case, having a history of synovial leishmaniasis explained the hand deformity and increased the likelihood of MCL diagnosis.

Leishmaniasis has been described as “a great imitator,” resembling a wide range of dermatoses, which explains why suspecting the disease is challenging for many physicians. Therefore, the differential diagnoses include a multitude of skin conditions, in addition to infectious, neoplastic, and rheumatological etiologies that have cutaneous manifestations. This stems from the fact that the way leishmaniasis manifests itself reflects how the leishmania parasite interacts with its host’s immune system [4]. Additionally, the possibility of developing superimposed cellulitis adds another layer of complexity, as culturing the purulent discharge can yield consistent bacterial growth, and the patient could be misdiagnosed and mistreated solely as a case of cellulitis [6]. It is therefore essential for physicians to be aware of the spectrum of disease presentations and its numerous gross appearances. In general, CL needs to be considered when a patient presents with skin lesions that have been present for weeks or months, changed slowly over time, and/or failed previous trials of treatment with antibiotic therapies. This is especially true for patients who have relevant personal and epidemiologic risk factors [4].

Assessment of risk factors is especially important when the differential is broad, as in our case. Our patient had two main factors pointing toward MCL as the most likely diagnosis. First, he has a history of leishmaniasis and is on prednisolone therapy for SLE. Leishmaniasis and its relapse have been documented with increased risk in patients on chronic immunosuppressive therapies [7,8]. The first case of CL reactivation in a patient on chronic systemic steroid therapy is probably the one reported by Tuon et al. (2007). Their patient was on prednisone 20 mg orally once daily for rheumatoid arthritis [9]. Second, our patient reported stopping miltefosine secondary prophylaxis a few months earlier. These two factors put relapsing MCL at the top of our differential.

Although MCL was readily suspected in this case, cutaneous SLE and cutaneous lymphoma (especially mycosis fungoides) were important differentials given the patient’s history of SLE and NHL, respectively. Another important differential diagnosis to consider with the described presentation is leprosy [10].

Ultimately, the diagnosis of MCL was confirmed by the PCR test, skin biopsy, and the patient’s positive response to therapy. Notably, the skin biopsy showed intense infiltration by inflammatory cells, but with scarce parasites. This is consistent with a form of CL termed “disseminated CL," which is usually contrasted to another form called “diffuse CL,” in which the lesions are not ulcerative and parasites are abundant in smears/biopsies [11,12]. However, there are no established therapeutic implications for this distinction [13].

There are many treatment options for leishmaniasis. However, evidence on the best choices and approaches is limited even for the more common forms [14,15]. We used systemic therapy with liposomal amphotericin B because the patient’s lesions were not localized; the exact species was unknown. Also, several large case series have demonstrated its efficacy in patients with mucosal involvement [16,17]. It has reported efficacy in disseminated CL as well, as demonstrated in the open clinical trial by Machado et al. (2015). The study assessed the cure rate at three months with doses starting from 17 mg/kg [18].

The patient had a positive response to the administered treatment. This was evident by the improvement noted in the skin lesions (Figure 2), the decrease in C-reactive protein level, and improved anemia (Table 1).

## Conclusions

Leishmaniasis is a common protozoal infection that could have rare and unusual presentations. The greatest challenge posed by this disease stems from the fact that it can mimic a wide range of dermatologic conditions, and as a result, it can be easily overlooked by the non-suspecting physician. Therefore, visual awareness of the spectrum of disease presentations is arguably the most challenging and important skill to acquire in the diagnosis and management of CL. This case represents a rare presentation of leishmaniasis in an immunocompromised patient with a history of multiple treated diseases and comorbidities, some of which constituted possible differentials for his presentation. 
